# Prenatal diagnosis of fetal skeletal dysplasia using targeted next-generation sequencing: an analysis of 30 cases

**DOI:** 10.1186/s13000-019-0853-x

**Published:** 2019-07-13

**Authors:** Yan Liu, Li Wang, Yi-Ke Yang, Ying Liang, Tie-Juan Zhang, Na Liang, Li-Man Yang, Si-Jing Li, Dan Shan, Qing-Qing Wu

**Affiliations:** 1 Department of Obstetrics, Beijing Obstetrics and Gynecology Hospital, Capital Medical University, Beijing, 100026 China; 2 Department of Ultrasound, Beijing Obstetrics and Gynecology Hospital, Capital Medical University, Beijing, 100026 China; 3 Department of Radiology, Beijing Obstetrics and Gynecology Hospital, Capital Medical University, No. 251 of Yaojia Yuan Street, Chaoyang District, Beijing, 100026 China

**Keywords:** Fetal skeletal dysplasia, Chromosomes, Targeted next-generation sequencing, Whole genome sequencing, Prenatal diagnosis

## Abstract

**Background:**

This study aims to provide genetic diagnoses for 30 cases of fetal skeletal dysplasia, and a molecular basis for the future prenatal diagnosis of fetal skeletal dysplasia.

**Methods:**

A total of 30 cases of fetal skeletal dysplasia detected with ultrasound between January 2014 and June 2017 were analyzed. Among these fetuses, 15 fetuses had local skeletal malformations, while 15 fetuses had short limb malformations. Samples of fetal umbilical cord blood, amniotic fluid, and/or aborted tissue were collected from all cases. Karyotyping, whole genome sequencing, and targeted next-generation sequencing of skeletal disease-related pathogenic genes were performed, as needed. Blood samples were taken from the parents for verification using Sanger sequencing.

**Results:**

Among the 30 cases of fetal skeletal dysplasia, two cases were diagnosed with trisomy 18. However, none of these cases were identified with any microdeletions or microreplications associated with skeletal dysplasia. Among the 28 chromosomally normal cases with fetal skeletal dysplasia, 21 cases were detected with mutations in genes related to skeletal diseases. Furthermore, collagen gene mutations were detected in six fetuses with short limb malformations, while heterozygous disease-causing mutations in the fibroblast growth factor receptor 3 (*FGFR3*) gene were detected in seven fetuses. The remaining fetuses carried mutations in other various genes, including tumor protein p63 (*TP63*), cholestenol delta-isomerase (*EBP*), cholinergic receptor nicotinic gamma subunit (*CHRNG*), filamin B (*FLNB*), and SRY-box 9 (*SOX9*). Three compound heterozygous mutations in *CHRNG*, *COL11A2* and *SOX9* were carried by phenotypically healthy parents.

**Conclusion:**

Targeted next-generation sequencing can significantly improve the prenatal diagnoses of fetal skeletal dysplasia, providing parents with more precision medicine, and improved genetic counseling.

## Background

Fetal skeletal dysplasia is an osteochondroblastic disease that has strong clinical heterogeneity, affecting approximately 2.4–4.5 of 10,000 births [1–4]. Although fetal skeletal dysplasia is associated with few chromosomal abnormalities, this disease is mostly associated with mutations in genes that regulate bone formation [5, 6]. At present, the prenatal diagnosis of fetal skeletal dysplasia mostly relies on ultrasound, X-ray and magnetic resonance imaging [7–9]. In 40–49% of cases with fetal skeletal dysplasia, ultrasound cannot differentiate among the different types of skeletal dysplasia. Hence, this has been merely used to identify severe lethal skeletal dysplasia [10–12]. In the 2010 revision of the Nosology and Classification of Genetic Skeletal Disorders, 456 conditions were classified into 40 groups defined by molecular, biochemical and radiographic criteria [13]. Among these conditions, 316 conditions were associated with mutations in one or more of 226 different genes, providing a basis for the molecular genetic diagnosis of fetal skeletal dysplasia.

Most previous studies have focused on specific genetic diagnoses for dyschondroplasia, osteogenesis imperfecta, and simple limb deformities [2, 14, 15]. The present study analyzed 30 cases of fetal skeletal dysplasia. The aim of the present study was to increase the scope of prenatal diagnoses and improve the genetic counseling offered to parents. In addition, the present study aimed to provide a theoretical basis for the early implementation of birth defect intervention and reproductive risk assessment.

## Methods

### Demographic features of cases

The present study comprised of 30 cases of fetuses diagnosed with skeletal abnormalities via ultrasound at the Obstetrics and Gynecology Hospital of Capital Medical University, Beijing, China between January 2014 and June 2017. Among these 30 cases, 15 cases were fetal skeletal malformations, while 15 cases were systemic skeletal dysplasias, which were characterized as short limb deformities. For the diagnosis of short limb deformity, two criteria must be simultaneously satisfied. First, the long bones of the extremities (i.e. the femur and humerus) must be shorter than the 5th percentile for fetuses of the same gestational age. In addition, the femur length (FL) to abdominal circumference (AC) ratio must be ≤1.6. Second, there must be ultrasonographic manifestations of abnormal bone morphology, such as long bone bending, angulation, fractures, “telephone receiver-shaped” changes, thoracic dysplasia, or changes in bone mineral density [10]. The present study was approved by the ethics committee of our hospital, and all parents of the fetuses provided a signed informed consent prior to prenatal diagnosis and sample collection.

### Sample collection

After routine disinfection, fetal umbilical cord blood puncture was performed, and 2 mL of blood was drawn from the umbilical vein into a vacuum blood collection tube containing ethylenediaminetetraacetic acid (EDTA). If no fetal blood sample could be obtained, two pieces of fetal muscle tissue (3 × 3 cm) containing the skin were removed after abortion. For all cases, 5 mL of venous blood was collected from both parents in vacuum blood collection tubes containing EDTA. All specimens were treated and stored at − 80 °C until use. Both the sources of the samples and methods of blood sampling were approved by the Institutional Ethics Committee of the Obstetrics and Gynecology Hospital of Capital Medical University. The informed consent forms were completed by the parents.

### Detection of fetal chromosomal abnormalities

Fetal amniotic fluid or umbilical cord blood samples were taken for chromosome G band karyotype analysis.

### Whole genome sequencing (WGS) to detect fetal microdeletions/microduplications

Umbilical cord blood punctures were performed on fetuses with abnormal skeletal abnormalities. Complete genomic DNA was extracted using a commercial DNA extraction kit (Puregene; Qiagen, Hilden, Germany) from either the umbilical cord blood, or muscle tissue samples. WGS with 20–30× coverage, combined with bioinformatics analysis, accurately localize microdeletions and microduplications ≥100 kb long. If ≥100 kb disease-related microdeletions are detected in fetal tissues, these results were verified in the parent samples using Sanger sequencing.

### Detection of variants in known genes related to congenital skeletal anomalies

If neither chromosomal abnormalities, nor disease-related microdeletions/microduplications were detected, the protein-coding regions and adjacent regions of 30 bp of known genes related to congenital skeletal anomalies were deeply sequenced using the targeted gene sequencing method. A special capture array (BGI, China) was used to detect the variants of the 363 genes involved in congenital skeletal anomalies. The overall sequencing coverage of the target regions was 97.81% for 20× depth of coverage in each of the chromosomes. After filtration by SAMtools (version 1.4), the sequencing data were mapped to the human genome (NCBI37/hg19) using the Burrows Wheeler Aligner software, and single nucleotide variants were identified using the SOAPsnp software (version 2.0). Then, the biological information was compared to databases, including ExAC, dbSNP, HapMap, 1,000 Genomes Asian, ESP6500, Cosmic, and HGMD. All variants were classified according to American College of Medical Genetics and Genomics recommended standards. After matching the inherent patterns of the disease, the pathogenicity of these loci were further determined by assessing the clinical symptoms and genetic data.

### Verification of gene mutations

If pathogenic mutations were detected in the fetal samples, these mutations were further verified in the parents by Sanger sequencing.

## Results

### Clinical features of cases

Among the 30 cases in the present study, 15 fetuses had local deformities (including varus deformities, finger/toe deformities, missing fingers/toes, and/or absence of upper/lower limbs), while 15 fetuses had systemic general skeletal dysplasia characterized by short limb deformities. All deformities were confirmed by postpartum clinical and pathological analysis (Table 1).

**Table 1:** General data for 30 cases of fetal skeletal dysplasia, including chromosome information and microdeletion/microduplication test results

No.	Ultrasound results	Gestation (weeks)	Chromosome	Micro-deletion/duplication results and significance
1	Right choroid plexus cyst and left foot inversion in the fetus	21	Trisomy 18	
2	Absence of radius in the upper limbs of the fetus, abnormal posture of both hands	20	Trisomy 18	
3	Continuously interrupted left upper lip, broken upper alveolar bone, possible small jaw, and absent fetal femur	25	46,XN	No abnormalities
4	Spinal fissure (isopathology), gastroschisis, double lung dysplasia, bipedal varus, left foot polydactyly	25	46,XN	arr Xq26.2(133,527,188-133,533,879)×1 There is a 6.6 Kb deletion in the Xq26.2 segment of the fetal X chromosome. This fragment spans exon 4 and exon 5 of the PHF6 gene and does not correlate well with the patient’s clinical phenotype.
5	Bending bilateral femur, tibia and fibula, fixed knee, internal crossed flexion, and fixed foot position	23	46,XN	No abnormalities
6	Cleft lip and palate, right kidney cystic dysplasia, bilateral foot fissure, and syndactyly, partially absent fingers of both hands	25	46,XN	No abnormalities
7	Fetal bilateral femoral angulation deformity, fetal heart ventricular septal defect	24	46,XN	46,XN,dup(7q11.21)(64,635,655-64,947,696) × 3,46,XN,del(11p11.12)(49,009,009-49,120,197) × 1,46,XN,del(17p12)(14,099,119-15,464,828) × 1 No clear pathogenic >100Kb microdeletions/microreplications
8	Right foot inversion, bilateral rocker bottom feet, scoliosis, spina bifida occulta	30	46,XN	No abnormalities
9	Fetal scoliosis, fetal bipedal varus, ventricular septal defect	26	46,XN	No abnormalities
10	Absence of bilateral ulna and radius, absence of bilateral humerus, foot inversion, abnormal wrist joints	24	46,XN	arr 2q24.3(166,914,464-166,920,459)×1 There is a 5.9Kb deletion in chromosome 2q24.3 on chromosome 2, which is not associated with a clinical phenotype.
11	Absence of bilateral humerus and left foot	26	46,XN	No abnormalities
12	Only two visible metacarpal bones on the left hand and part of distal phalanx on the lateral and medial sides	26	46,XN	No abnormalities
13	Left foot inversion, absence of right lower limb	26	46,XN,21cenh+	arr 5q35.1(170,405,440-171,071,061)×3 There is a repeat of 665 Kb fragment in the 5q35.1 segment of fetus chromosome 5, which includes 4 OMIM genes such as NPM1. The correlation with clinical phenotype is not high.
14	Absence of fetal fibula, foot inversion, partial absence of phalanges, absence of fingers, hand cleft deformity	22	46,XN	No abnormalities
15	Fetal sirenomelia	17	46,XN	No abnormalities
16	Short limbs - incomplete osteogenesis?	23	46,XN	No abnormalities
17	Short limbs - incomplete osteogenesis	22	46,XN	No abnormalities
18	Short limbs	22	46,XN	No abnormalities
19	Short fetus limbs: cartilage hypoplasia?	22	46,XN	No abnormalities
20	Short limbs, spine and vertebral ossification are not obvious: cartilage hypoplasia?	16	46,XN	No abnormalities
21	Short limbs, ventricular septal defect	15	46,XN	No abnormalities
22	Abnormal long bones in fetal limbs, narrow chest	24	46,XN	No abnormalities
23	Short fetal limbs (chronic dysgenesis)	25	46,XN	No abnormalities
24	Short limbs (cartilage hypoplasia)	25	46,XN	arr 19p13.2(11,135,293-11,139,948)x, There is a deletion of 4.6 Kb fragment in the 19p13.2 segment of chromosome 19 of the fetus and there is no correlation with the patient’s clinical phenotype.
25	Short limbs	21	46,XN	No abnormalities
26	Short limbs	21	46,XN	No abnormalities
27	Short limbs	27	46,XN	46,XN,dup(2p11.2)(87,384,213-87,862,105) × 3, Polymorphism
28	Short limbs	25	46,XN	No abnormalities
29	Achondroplasia	15	46,XN	No abnormalities
30	Uneven arrangement of fetal spine, short limbs, bilateral foot inversion, small mandibular, left ventricular punctate strong echo, ventricular septal defect	23	46,XN	Both chromosomes 1 and 16 have microduplications. Dup(Xq27.1)(139,911,843-140,072,771) × (2~3) Polymorphism

*arr* microarray, *dup* duplication, *del* deletion

### Skeletal chromosomal abnormalities and microdeletions/microduplications

All 30 cases of fetal skeletal dysplasia were tested for chromosomal abnormalities. Trisomy 18 was detected in two cases of fetal local skeletal malformation (cases 1–2, Table 1). However, neither chromosomal abnormalities, nor pathological microdeletions/microduplications related to skeletal dysplasia were identified in the remaining 28 cases.

### Sequencing and verification of hereditary bone disease in 13 cases of fetal local skeletal malformation

Among the 13 cases of fetal local skeletal malformation, in which no chromosomal abnormalities were identified, seven cases (cases 9–15) had no pathogenic mutations, while six cases (cases 3–8) had mutations in known genes associated with bone diseases (Table 1). In three of these cases (cases 3–5), the mutations were of unknown clinical significance, while in the remaining three cases (cases 6–8), the mutations were considered as causative.

### Detection of hereditary bone disease mutations using targeted gene sequencing and validation using sanger sequencing in 15 cases of systemic skeletal dysplasia (short extremities)

Collagen gene mutations were detected in six cases (cases 16–21). The clinically unexplained mutation was only identified in case 20: c.2419G > A (p.Gly807Arg) in the *COL2A1* gene. For the remaining cases, the mutations were known to cause the disease.

FGFR3 mutations were detected in seven cases diagnosed with short limbs (cases 23–29). Four of these cases (cases 23–26) carried the same mutation: c.742C > T (p.Arg248Cys) in the *FGFR3* gene. Each of the remaining three cases carried a single unique mutation: c.1144G > A (p.Gly382Arg), c.1124A > G (p.Tyr375Cys), or c.2426G > C (p.X809S,101). Sanger sequencing confirmed that none of these mutations were carried by any parent.

In case 22, a heterozygous mutation in the emopamil binding protein (*EBP*) gene (NM_006579.2) was detected (C.440G > A, p.Arg147His). In case 30, a clinically significant heterozygous mutation in the filamin B (*FLNB)* gene was detected (c.475A > C, p.Thr159Pro). For both cases, the parents were not the carriers, suggesting that these were new fetal mutations. The mother of case 22 has subsequently given birth to a healthy baby boy (Table 2).

**Table 2:** Identification of genes associated with skeletal diseases in 30 cases of fetal skeletal dysplasia

No.	Ultrasound results	Inheritance	Bone gene-encoded	Reference sequence	Nucleotide change/mutation	Amino-acid change	Genetic subregion	Heterogeneity	Chromosomal Loci	Mutation type	Sanger verification		Next pregnancy/child
											Paternal	Maternal	
1	Right choroid plexus cyst and left foot inversion in the fetus		Trisomy 18										Delivered a healthy child
2	Absence of radius in the upper limbs of the fetus, abnormal posture of both hands		Trisomy 18										Delivered a healthy child
3	Continuously interrupted left upper lip, broken upper alveolar bone, possible small jaw, and absent fetal femur	AD	TBX4	NM_018488.2	c.1200G > T	p.Glu400Asp	EX8E	Het	Chr17:59560439	VUS	N	Het	
4	Spinal fissure (isopathology), gastroschisis, double lung dysplasia, bipedal varus, left foot polydactyly	AD	TNNT3	NM_006757.3	c.88G > A	p.Ala30Thr	EX7	Het	Chr 11:1950355	VUS	Het	N	Delivered a healthy child
5	Bending bilateral femur, tibia and fibula, fixed knee, internal crossed flexion, and fixed foot position	AD	SOX9	NM_000346.3	c.344G > C	p.Trp115Ser	EX1	Het	Chr17:70117876	VUS	N	N	Delivered a healthy child
6	Cleft lip and palate, right kidney cystic dysplasia, bilateral foot fissure, and syndactyly, partially absent fingers of both hands	AD	TP63	NM_003722.4	c.952C > T	p.Arg318Cys	EX7	Het	Chr3:189585691	Pathogenic	N	N	
7	Fetal bilateral femoral angulation deformity, fetal heart ventricular septal defect	AR	POR	NM_000941.2	c.1370G > A	p.Arg457His	EX12	Het	Chr7: 75614497	Pathogenic	N	Het	Angular femoral deformity found at the 15th week of pregnancy, pregnancy terminated
					c.744C > G	p.Tyr248*	EX8	Het	Chr7: 75611554	Pathogenic	Het	N	
8	Right foot inversion, bilateral rocker bottom feet, scoliosis, spina bifida occulta	AR	CHRNG	NM_005199	C.13C > T	p.Q5X,513	EX1	Het	Chr2	Pathogenic	N	Het	Same abnormality as the previous pregnancy, undergoing PGD
					C.202C > T	p.R68X,450	EX3	Het	Chr2	Pathogenic	Het	N	
9	Fetal scoliosis, fetal bipedal varus, ventricular septal defect		No abnormalities										
10	Absence of bilateral ulna and radius, absence of bilateral humerus, foot inversion, abnormal wrist joints		No abnormalities										
11	Absence of bilateral humerus and left foot		No abnormalities										
12	Only two visible metacarpal bones on the left hand and part of distal phalanx on the lateral and medial sides		No abnormalities										
13	Left foot inversion, absence of right lower limb		No abnormalities										
14	Absence of fetal fibula, foot inversion, partial absence of phalanges, absence of fingers, hand cleft deformity		No abnormalities										Delivered a healthy child
15	Fetal sirenomelia		No abnormalities										Delivered a healthy child
16	Short limbs - incomplete osteogenesis?	AD	COL1A1	NM_000088.3	c.1678G > A	p.Gly560Ser	EX25	Het	Chr17: 48268739	Pathogenic	N	N	
17	Short limbs - incomplete osteogenesis	AD	COL1A2	NM_000089.3	c.1774G > A	p.Gly592Ser	EX31	Het	Chr7: 94045726	Pathogenic	N	N	
18	Short limbs	AD	COL1A2	NM_000089.3	c.1072G > A	p.Gly358Ser	EX20	Het	Chr7: 94039590	Pathogenic	N	N	Delivered a healthy child
19	Short fetus limbs: cartilage hypoplasia?	AD	COL2A1	NM_001844.4	c.3013G > A	p.Gly1005Ser	CDS44	Het	Chr12:48371891	Pathogenic	N	N	
20	Short limbs, spine and vertebral ossification are not obvious: cartilage hypoplasia?	AD	COL2A1	NM_001844.4	c.2419G > A	p.Gly807Arg	EX37	Het	Chr12:48375170	VUS	N	N	
21	Short limbs, ventricular septal defect	AD/ AR	COL11A2	NM_080680.2	c.966dupC	p.Thr323Hisfis*19	EX8	Het	Chr6:33152074	Pathogenic	Het	N	Undergoing PGD
					c.1773 + 8 T>A	–	IVS19	Het	Chr6:33146204	VUS	N	Het	
22	Abnormal long bones in fetal limbs, narrow chest	XD	EBP	NM_006579.2	c.440G>A	p.Arg147His	EX4	Het	ChrX:48385644	Pathogenic	N	N	Delivered a healthy boy
23	Short fetal limbs (chronic dysgenesis)	AD	FGFR3	NM_001163213.1	c.742C > T	p.Arg248Cys	EX7	Het	Chr4:1803564	Pathogenic	N	N	Delivered a healthy child
24	Short limbs (cartilage hypoplasia)	AD	FGFR3	NM_001163213.1	c.742C > T	p.Arg248Cys	EX7	Het	Chr4:1803564	Pathogenic	N	N	
25	Short limbs	AD	FGFR3	NM_001163213.1	c.742C > T	p.Arg248Cys	EX7	Het	Chr4:1803564	Pathogenic	N	N	
26	Short limbs	AD	FGFR3	NM_001163213.1	c.742C > T	p.Arg248Cys	EX7	Het	Chr4:1803564	Pathogenic	N	N	
27	Short limbs	AD	FGFR3	NM_001163213.1	c.1144G > A	p.Gly382Arg	EX9	Het	Chr4:1806119	Pathogenic	N	N	
28	Short limbs	AD	FGFR3	NM_001163213.1	c.1124A > G	p.Tyr375Cys	EX9	Het	Chr4:1806099	Pathogenic	N	N	
29	Achondroplasia	AD	FGFR3	NM_001163213.1	c.2426G > C	p.X809S,101	EX18	Het	Chr4:1806099	Pathogenic	N	N	
30	Uneven arrangement of fetal spine, short limbs, bilateral foot inversion, small mandibular, left ventricular punctate strong echo, ventricular septal defect	AD	FLNB	NM_001164317.1	c.475A > C	p.Thr159Pro	EX2	Het	Chr3:58062955	VUS	N	N	

*Het* heterogeneity, *Pathogenic* known disease-causing mutation, *VUS* Mutations of unknown clinical significance, *N* normal, *AD* autosomal dominant, *AR* autosomal recessive, *PGD* preimplantation genetic diagnosis

## Discussion

### Prenatal diagnosis of local skeletal dysplasia

**Fig. 1:**
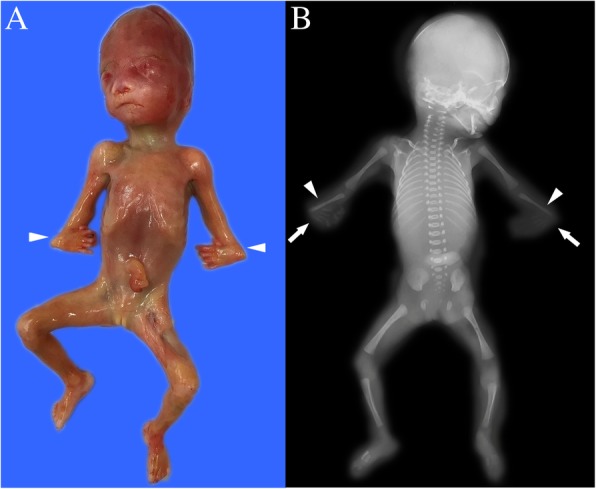
Case 2: The fetus with trisomy 18 and the absence of the radius. **a** Gross anatomy of the aborted fetus, showing the bilateral wrist flexion (arrowheads). **b** X-ray image of the aborted fetus, showing the bilateral bone defects (arrowheads) and bilateral wrist flexion (arrows)

Different types of chromosomal abnormalities complicate the wide, diverse variety of skeletal abnormalities [16]. For example, fetuses carrying trisomy 13, trisomy 18, or even trisomy 21 (Down’s syndrome) may have abnormal skeletal development. In the present study, two fetuses with trisomy 18 (cases 1 and 2) exhibited chromosomal abnormalities in local bone lesions, suggesting that the screening for chromosomal abnormalities remains vital when skeletal lesions are observed (Fig. 1).

The WGS revealed no significant copy number variations in the 13 cases of local fetal skeletal abnormalities (cases 3–15, Table 1). In addition, no mutations of skeletal pathogenic genes were identified in the seven cases (cases 9–15, Table 2). In contrast to osteodystrophy, local skeletal abnormalities are regulated by other factors in addition to hereditary genes. However, these additional factors require further study.

In the six fetuses with skeletal lesions (cases 3–8), skeletal gene mutations were detected with targeted gene sequencing (Table 2). In three of these cases (cases 3–5), the fetuses carried heterozygous mutations of unclear clinical significance. Both case 3 (femoral absence, micrognathia, and cleft lip and palate) and case 4 (spinal fracture, ventral fissure, introversion, and left foot polydactyly) carried clinically unexplained heterozygous mutations. The fetus in case 3 carried TBX4-induced small zygomatic complexes, while the fetus in case 4 carried TNNT3-related distal type 2B. Both of these are autosomal dominant genetic diseases. However, the Sanger sequencing suggested that one of the healthy parents carried the mutation. Hence, these two mutations could not explain the reason of case 3 and 4.

In case 5, the fetus carried a clinically unexplained heterozygous mutation in the *SOX9* gene. *SOX9*-related trunk dysplasia is an autosomal dominant genetic disease, and in such diseases, heterozygous mutations can induce a disease phenotype. In this case, the Sanger sequencing indicated that neither of the parents were mutation carriers. Hence, this mutation was considered de novo and likely pathologic. Notably, the mother became naturally pregnant again and gave birth to a healthy baby boy.

In the remaining three cases (cases 6–8), known disease-causing mutations were detected in the fetuses. The available information about these mutations was useful in fetal disease diagnosis and in the subsequent genetic counseling for the parents.

In case 6 (fetal cleft lip and palate, right kidney cystic hypoplasia, bilateral foot fissure, lateral toe, and abnormal hand development), the fetus carried a mutation in the *TP63* gene, which was correlated to ectrodactyly, ectoderm dysplasia, and cleft lip/palate syndrome-3 (EEC3). EEC3 is an autosomal dominant genetic disease, and in such diseases, heterozygous mutations can cause the disease. Alves et al. [17], Hydern et al. [18] and Clements et al. [19] all reported several mutations of the *TP63* gene in families of EEC3. The Sanger sequencing revealed negative results for both parents of case 6, and this mutation was thereby considered novel in the fetus. The subsequent genetic counseling advised the parents to continue to conceive naturally.

In case 7 (angular femoral deformity and ventricular septal defect), two compound heterozygous causative mutations in the *POR* gene were detected: c.1370G > A (p.Arg457His) and c.744C > G (p.Tyr248*), which were associated with the Antley-Bixler syndrome. The Antley-Bixler syndrome is an autosomal recessive disease, and this type of homozygous or compound heterozygous mutation can induce a disease phenotype. The mutation c.1370G > A (p.Arg457His) has been detected in patients diagnosed with Antley-Bixler syndrome [20, 21]. The other mutations have also been reported in recent years [22, 23]. The mutation c.744C > G (p.Tyr248*) is a nonsense mutation that prematurely terminates the encoding of the POR protein at amino acid 248 (the unmutated protein is 680 amino acids long). Although the specific mutation identified in the present study has not yet been reported in literature, previously reported nonsense mutations after position 248 were deleterious. POR gene mutations c.1370G > A and c.744C > G have been considered disease-causing in their compound heterozygous forms. In the present study, the Sanger sequencing verified that both parents were carriers of this disease-causing mutation. The parents chose to conceive naturally at 6 months after pregnancy termination. However, the pregnancy was terminated at the 15th week of the second pregnancy, because the ultrasound examination revealed bilateral femoral angulation again in the fetus. Compound heterozygous POR gene mutations c.1370G > A and c.744C > G were verified on the analysis of the aborted tissues. The parents conceived naturally for the third time. The ultrasound results were normal, and the amniotic fluid analysis indicated that the POR gene mutation was absent. The mother eventually delivered a healthy baby girl.

In case 8 (two-sided rocker bottom feet, scoliosis, and spina bifida occulta), compound heterozygous mutations were detected in the *CHRNG* gene. The fetus was diagnosed with both double-curve scoliosis and varus, and both parents were carriers of this disease-causing mutation. These mutations had an autosomal recessive inheritance pattern. Hence, both parents presented with a normal phenotype. The final fetal diagnosis was Escobar variant of multiple pterygium syndrome (EVMPS). The parents are presently trying to conceive with PGD.

### Prenatal diagnosis of systemic skeletal dysplasia

In the present study, no chromosomal abnormalities were identified in any of the cases of systemic skeletal dysplasia that manifested in short limb deformities (Table 1). The targeted gene sequencing identified several *disease-causative mutations* in known genes related to the disease (Table 2).

**Fig. 2:**
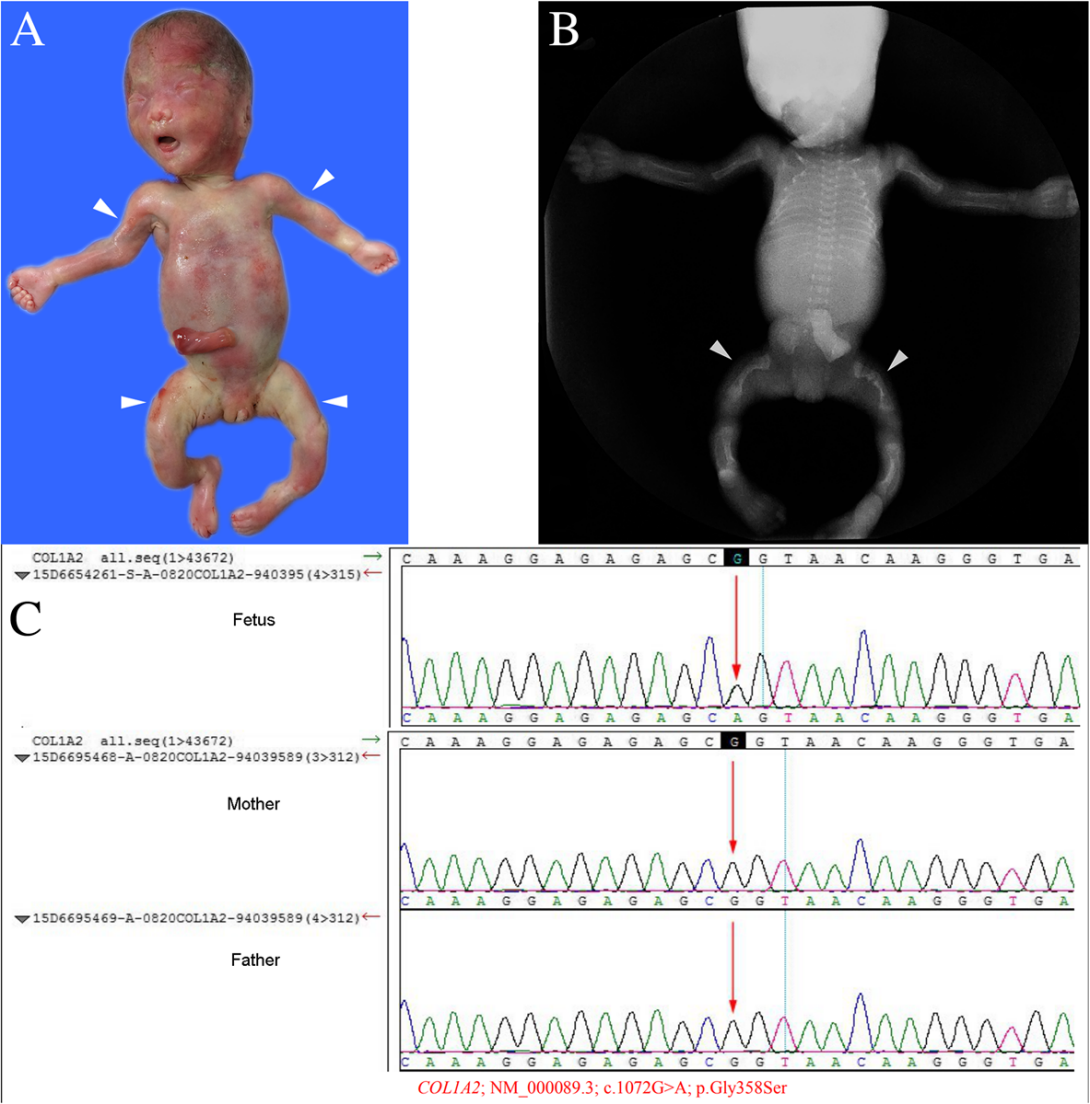
Case 18: The fetus with osteogenesis imperfecta type II with collagen gene mutation (COL1A2). **a** Gross anatomy of the aborted fetus, showing the short arms and bending short legs (arrowheads). **b** X-ray images of the aborted fetus, showing the short and bent femurs, both with fractures (arrowheads). **c** The fetus carried the c.1072G > A (p.Gly358Ser) mutation in the COL1A2 gene. The Sanger verification revealed that this mutation was new, and was not carried by the parents

Collagen is an indispensable component of bone tissue found in the extracellular matrix. Mutations in the collagen gene may lead to insufficient collagen production. There are substantial differences in the severity of skeletal abnormalities caused by different types of collagen mutations. Mutations in collagen genes were detected in cases 16–21 in the present study. Three fetuses (cases 16–18) were diagnosed with osteogenesis imperfecta based on prenatal ultrasound, gross postnatal pathology, and X-ray examination (Fig. 2). Heterozygous mutations in *COL2A1* were found in the other two fetuses (cases 19 and 20) diagnosed with achondrogenesis type II.

In case 16, a heterozygous causative mutation (c.1678G > A, p.Gly560Ser) in *COL1A1* was detected in the fetus. In cases 17 and 18, two known pathogenic mutations were detected in the *COL1A2* gene: c.1774G > A (p.Gly592Ser) and c.1072G > A (p.Gly358Ser). These mutations have been reported to be pathogenic mutations associated with osteogenesis imperfecta [24–28]. However, none of these mutations were detected in the parents, suggesting that these mutations are novel fetal mutations.

Approximately 90% of osteogenesis imperfecta cases are due to causative variants in the *COL1A1* and *COL1A2* genes, which result in abnormal collagen I fibrils formation, while the remaining 10% of cases are associated with recessive variants of known or yet to be discovered genes [24, 25].

**Fig. 3:**
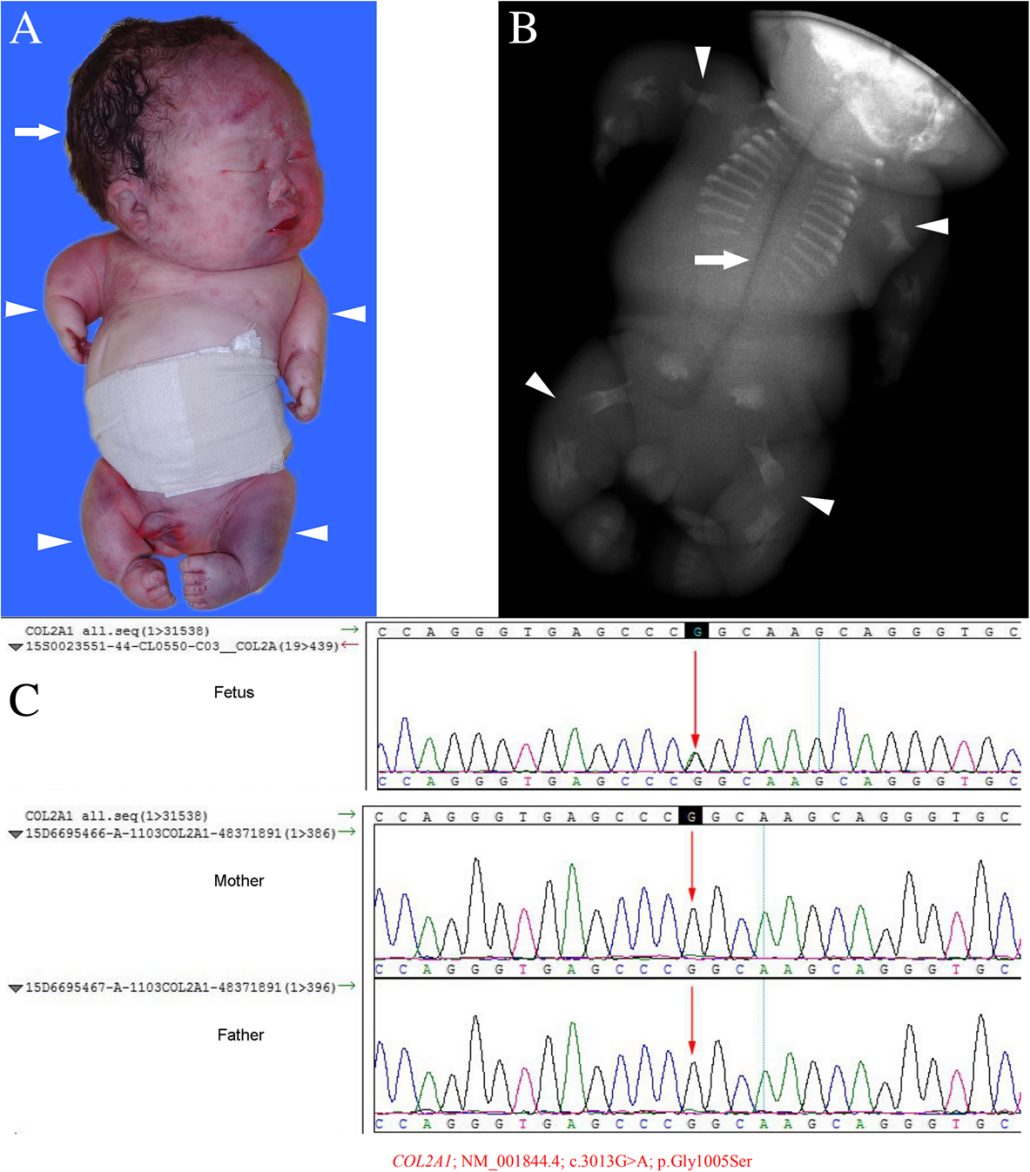
Case 19: The fetus with achondrogenesis. **a** Gross anatomy of the aborted fetus, showing the markedly short arms and legs (arrowheads), with a giant skull (arrow). **b** X-ray images of the aborted fetus, showing the severe short limb (arrowheads), and the defective ossification of the spine, sciatic, pubic and iliac bone (arrow). **c** The fetus carried the c.3013G > A (p.Gly1005Ser) mutation in the *COL2A1* gene. The Sanger verification revealed that this mutation was new, and was not carried by the parents

Heterozygous mutations in *COL2A1* were found in two fetuses (cases 19 and 20) diagnosed with achondrogenesis type II (Fig. 3). Mutations in *COL2A1* disrupt the Gly-XY motif necessary for the formation of a triple helix structure, resulting in type II collagen over-modification, cellular retention and decreased secretion [29, 30]. All these collagen-related mutations were new in the fetuses, and the parents were not mutation carriers. The parents were advised to continue natural conception.

Case 21 was eventually diagnosed with fibrocartilage hyperplasia type II, which was caused by mutations in the *COL11A2* gene. The two *COL11A2* mutations identified in the present study have only been previously identified once [31]. These mutations revealed autosomal recessive/dominant inheritance. The Sanger sequencing verified that these phenotypically normal parents were mutation carriers. These parents were trying to conceive again with PGD.

Fibroblast growth factors (FGFs) play an important role in endochondral osteogenesis and intramembranous osteogenesis. Cells generally aggregate in several areas on osteophyte growth plates, and in the proximal dormant area, chondrocytes proliferate. Then, the chondrocytes differentiate into primary hypertrophic chondrocytes, and gradually become mature hypertrophic chondrocytes. The proliferation, differentiation and apoptosis of chondrocytes are regulated by FGF/FGFR signaling. For example, the interaction between FGF18 and FGFR3 inhibits chondrocyte proliferation. Mutations in the *FGFR3* gene can increase the extracellular/tyrosine kinase domain activity of the receptor, stimulating the signaling pathways that induce the expression of extracellular signal-regulated kinase 1/2, and the signal transducers and activators of transcription protein 1 (STAT1), which leads to the arrest of chondrocyte proliferation and chondrocyte apoptosis .

**Fig. 4:**
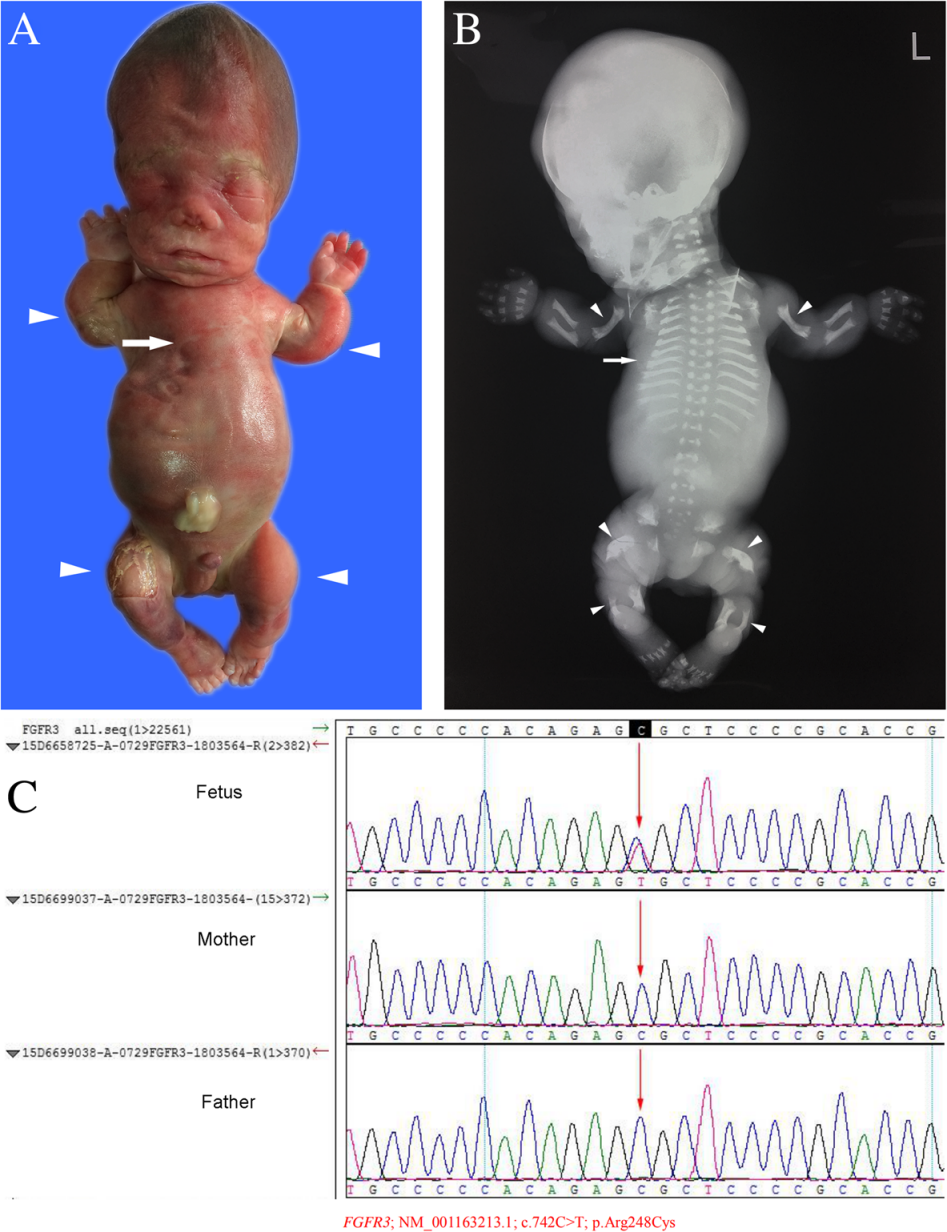
Case 26: The fetus with fatal achondroplasia and fibroblast growth factor receptor 3 (FGFR3) gene mutations. **a** Gross anatomy of the aborted fetus, showing the markedly short arms and legs (arrowheads), with a narrow chest (arrow). **b** X-ray images of the aborted fetus, showing the “telephone receiver” appearance of the femurs and humeri, the malformation of the metaphyseal (crateriform) (arrowheads), and the narrow thorax (arrow). **c** The fetus carried the c.742C > T (p.Arg248Cys) mutation in the *FGFR3* gene. The Sanger verification revealed that this mutation was new, and was not carried by the parents

In the present study, seven fetuses with achondroplasia (cases 23–29) were further examined with a combination of ultrasound, postnatal gross pathology, and X-ray. Based on these examinations, six of the fetuses were diagnosed with clinically fatal cartilage hypoplasia. The subsequent genetic analysis confirmed the fatal cartilage hypoplasia type I in six fetuses. Cases 23–26 carried an identical mutation (c.742C > T, p.Arg248Cys), which is a common pathogenic mutation associated with lethal achondroplasia (Fig. 4). In 2015, Barkova et al. [2] reported that eight of 20 patients with lethal dysplasia type I carried the c.742C > T mutation in *FGFR3*. Similar findings were reported in 2001 by Chen et al. [32] and in 2014 by Cho et al. [33]. The mutation c.1124A > G (p.Tyr375Cys) in the *FGFR3* gene is also a common pathogenic mutation that causes lethal dwarfism type I. The study conducted by Rousseau et al. [34] revealed that eight of 26 (30.7%) cases of fatal dwarfism type I carried the c.1124A > G (i.e. c.1118A > G in the article) mutation in *FGFR3*. However, Xue et al. [35] reported that the frequency of these *FGFR3* mutations was 23.7% (41 of 173 cases) in cases of fatal dwarfism type I. Another *FGFR3* mutation, c.2426G > C (p.X809S,101), was identified in the present study, which has not previously been reported. This was a missense mutation that led to the false extension of protein translation. In case 27, the fetus was diagnosed with achondroplasia. Furthermore, 99% of all achondroplasia cases are caused by mutations in the *FGFR3* gene, and c.1144G > A (p.Gly382Arg) is the most common pathogenic mutation. The missense mutation c.1144G > A (p.Gly382Arg) is identical to c.1138G > A (p.Gly380Arg) (different transcripts). In 1995, the study conducted by Bellus et al. [36] revealed that 187 of 193 (96.9%) cases of achondroplasia are caused by the mutation c.1138G > A. For case 27, since the parents did not carry the mutation, it was considered a new mutation in the fetus. After genetic counseling, the parents were advised to continue to conceive naturally.

The mutation c.475A > C (p.Thr159Pro) in the *FLNB* gene carried by the fetus in case 30 is a missense mutation, which changes the amino acid at position 159 from threonine to proline. *FLNB*-related osteogenesis imperfecta type I/Larsen syndrome is an autosomal dominant disease. The sequence verification confirmed that neither of the parents carried the mutation, indicating that this was a novel mutation in the fetus. Therefore, the parents were advised to continue to conceive naturally.

In case 22, ultrasound revealed the abnormal development of long bones of the limbs (the length of the long bones was less than 1%), thick metaphysis in the right lower limb, irregular vertebral arrangement, and a narrow and small thorax in 24 weeks of gestation. The fetus carried a heterozygous mutation in the *EBP* gene (C.440G > A, p.Arg147His). Herman et al. investigated the mutations in 26 female patients with suspected CDPX2. Among these 26 patients, 22 had EBP mutations. Among these 22 mutations, 13 mutations were de novo [37]. The *EBP* gene was located on the short arm of the X chromosome (Xp11.22-p11.23), and the mutations in this gene can lead to the accumulation of 8-dehydrocholesterol and 8(9)-cholesterol in plasma, the skin and other tissues, resulting in a wide range of abnormalities [37, 38]. In the present study, a heterozygous EBP mutation (a known causative mutation; c.440G > A [p.Arg147His]) was the cause of CDPX2, and is a missense mutation (arginine to histidine). The Sanger test revealed that the patient’s parents were non-carriers, indicating that the mutation of the fetus occurred de novo. Whittock et al., Has et al. and Cañueto et al. have previously reported cases related to this mutation site. [39–41]. However, neither of the parents carried the mutation. In the present study, the 27-week-old female fetus presented with markedly short bones, ankle joint contracture, markedly asymmetric short lower limbs, and a flat face and nose bridge. The severity of the phenotype was considered to be related to X chromosome inactivation, which is also known as lyonization.

However, although targeted exome capture and sequencing have shown great advantages in disease gene identification and molecular diagnosis, some problems still needs to be immediately resolved. Fox example, exome sequencing focuses on the sequencing of exon regions. Thus, from the genome level, the information obtained was obviously incomplete. Furthermore, information for promoter regions, enhancer regions and microRNA coding regions were certainly missed. Second, a large amount of data was obtained after exome sequencing. The best method to perform an in-depth and accurate analysis of these data is the largest challenge faced at present by researchers worldwide. The deep mining of data needs to start from many aspects and perspectives, including studies at the transcription level, bioinformatics analysis, and functional genomics studies.

## Conclusion

In summary, the results of the present study suggest that the application of targeted gene sequencing technology can significantly improve the prenatal diagnosis of systemic skeletal abnormalities, allowing for a more comprehensive and useful prenatal genetic counseling guidance for parents. Furthermore, the present study provides a theoretical basis for early intervention birth defect diagnoses and the assessment of fetal risk associated with subsequent pregnancies. In addition, the present study also provides further useful information for the continued development of skeletal dysplasia treatments based on target genes [32]. To date, mutations in 363 genes are known to be associated with more than 300 common skeletal dysplasias in humans [10]. However, genetic basis remains unknown in many additional skeletal diseases, especially local skeletal lesions, suggesting that new genes or non-genetic factors may cause these diseases.

## Data Availability

All data generated or analysed during this study are included in this published article [and its supplementary information files].

## Abbreviations

AC: Abdominal circumference
CHRNG: Cholinergic receptor nicotinic gamma subunit
EBP: Cholestenol delta-isomerase
EDTA: Ethylenediaminetetraacetic acid
EEC3: Ectrodactyly, ectoderm dysplasia, and cleft lip/palate syndrome-3
EVMPS: Escobar variant of multiple pterygium syndrome
FGFR3: Fibroblast growth factor receptor 3
FGFs: Fibroblast growth factors
FL: Femur length
FLNB: Filamin B
SOX9: SRY-box 9
STAT1: Signal transducers and activators of transcription protein 1
TP63: Tumor protein p63
WGS: Whole genome sequencing
